# System-wide immunoregulation by polyvalent IgG: integrating transcriptomic, miRNA, and proteomic landscapes

**DOI:** 10.1186/s12967-026-08289-6

**Published:** 2026-05-30

**Authors:** João Vitor da Silva Borges, Lhays Ozório Passos, Nicolle Rakanidis Machado, Lais Alves do Nascimento, Beatriz Oliveira Fagundes, Isabela Siuffi Bergamasco, Anna Luisa Baratelli Moreira, Natali Espasiani Cilento, Sabri Saeed Sanabani, Jefferson Russo Victor

**Affiliations:** 1 Medical School, Santo Amaro University (UNISA), Sao Paulo, Brazil; 2 Post Graduation Program in Health Sciences, Santo Amaro University (UNISA), Sao Paulo, Brazil; 3 Laboratory of Medical Investigation LIM-56, Division of Dermatology, Medical School, University of São Paulo, 500 Av. Dr. Enéas Carvalho de Aguiar, São Paulo, 05403-000 Brazil; 4 Laboratory of Medical Investigation LIM-03, Clinics Hospital, Medical School, University of São Paulo, São Paulo, Brazil

**Keywords:** Polyvalent IgG (pIgG), Intravenous immunoglobulin (IVIg), Idiotypic network, Immune regulation, Transcriptomics, microRNA, Proteome microarray, Lymphocyte modulation, Th17 cells, Regulatory T cells, γδ T cells, IL-10–producing B cells, TRIM21, Antibody repertoires, Epitope profiling

## Abstract

**Background:**

Polyvalent IgG (pIgG/IVIg), derived from thousands of donors, exerts broad immunomodulatory effects; however, its direct cellular targets and genome-wide regulatory impacts remain incompletely defined.

**Methods:**

Peripheral blood mononuclear cells (PBMCs) from 20 healthy donors were cultured for 72 h with pIgG (100 µg/mL) or mock conditions. Cell viability, phenotype, memory subsets, chemokine receptors, and intracellular cytokines were assessed by flow cytometry. Differential gene expression, miRNA/sRNA profiles, and pathway enrichments were analyzed by RNA-seq and small-RNA sequencing. Proteome-wide IgG binding was mapped using human proteome microarrays across lymphoid subsets, and pathogen-derived linear epitope recognition was evaluated using a 4,345-epitope infectious-disease microarray.

**Results:**

pIgG preserved PBMC viability but significantly modulated immune function: it reduced IL-17⁺ CD4⁺ T cells and tissue-resident memory T cells, decreased IL-4⁺ CD8⁺ T cells, enhanced IFN-γ⁺ γδ T cells, downregulated CCR5 and CCR6, and expanded IL-10⁺ B cells while reducing IL-13⁺ and IL-17⁺ B cells. Transcriptomic analysis revealed 4,820 upregulated and 2,160 downregulated genes, with enrichment of MHC class II and TCR-signaling pathways and selective modulation of cytokine, chemokine, CD, and HLA genes. Innate sensors—including TLR4, TLR8, and NLR4—were downregulated. Small-RNA analysis identified 19 differentially expressed miRNAs and 79 novel sRNAs, indicating epigenetic remodeling. Proteome-wide profiling detected ~ 130 IgG-recognized proteins per lymphocyte subset and 180 shared targets enriched in cytosolic, vesicular, and endocytic pathways. Pathogen-epitope profiling identified 948 recognized sequences from 56 viruses, 21 bacteria, and 12 parasites.

**Conclusions:**

pIgG functions as a multilayered immunoregulator that attenuates Th17-associated inflammation, promotes IL-10–mediated regulatory circuits, and modulates receptor signaling and antigen-processing pathways. These findings underscore its potential for broad therapeutic immunomodulation.

**Supplementary Information:**

The online version contains supplementary material available at 10.1186/s12967-026-08289-6.

## Introduction

Polyvalent IgG (pIgG) for therapeutic use, administered intravenously and commonly referred to as intravenous immunoglobulin (IVIg), is produced from plasma pooled from thousands of healthy donors [1]. It therefore represents a broad sampling of the human IgG antibody repertoire, predominantly of the IgG1 subclass [2].

For decades, studies have demonstrated that pIgG can mediate complex immunological interactions in vivo. These include activation of the complement system, modulation of cytokine production, regulation of the idiotypic antibody network, modulation of receptor expression, and activation of lymphocytes and antigen-presenting cells [3]. More specifically, pIgG has been shown to suppress interferon-γ (IFN-γ) production by peripheral blood mononuclear cells (PBMCs) and cord blood cells [4, 5]. Similar effects have been reported on the production of IL-12, IL-6, and TNF, as well as on lymphocyte activation and apoptosis [4, 6–9]. Moreover, pIgG has been implicated in the suppression of T cell–mediated allogeneic responses [10], suggesting that idiotypic recognition of T cell clonal receptors contributes, at least in part, to its immunoregulatory mechanisms.

pIgG has also proven beneficial in preventing recurrent spontaneous abortions [11], a condition linked to autoantibody production [12–14]. This finding reinforces the hypothesis that transferred pIgG can engage in idiotypic interactions with human lymphocytes. Additional clinical studies have described the therapeutic potential of pIgG in severe childhood asthma [15], atopic dermatitis (AD), where clinical improvement was observed [16, 17], and pemphigus vulgaris, in which complete remission was reported [18]. Although such evidence highlights the immunomodulatory potential of pIgG/IVIg, the precise molecular targets underlying these effects remain incompletely understood [19–22].

An important consideration in this context is that pIgG formulations are derived from thousands of donors, inevitably introducing intra- and inter-batch variability. This heterogeneity contributes to inconsistencies across published findings and complicates efforts to define specific lymphocyte receptors or molecular targets recognized by pIgG [23, 24]. As a result, all approaches using pooled formulations are subject to potential biases stemming from donor diversity.

To address this challenge, mechanistic studies of pIgG have largely followed two main strategies: the investigation of crystallizable fragments (Fc), which mediate receptor-dependent interactions, and antigen-binding fragments (Fab), which recognize antigens through their variable regions. Since the early 1990s, Fc fragments have been suggested to mediate at least some of the in vivo effects of pIgG [25], likely through engagement of Fcγ receptors (FcγRs) expressed on multiple immune cell subsets. Because of their relatively limited diversity, Fc-mediated effects can be more readily investigated in vitro. However, in vivo studies are strongly influenced by host genetic background and dosage, contributing to inconsistent findings in the literature [26].

By contrast, Fab fragments exhibit considerable variability, representing an extensive set of idiotypes contributed by each donor. This complexity poses major challenges for elucidating Fab-mediated interactions. Since the first demonstrations of therapeutic efficacy of pIgG formulations in the early 1980s, it has been hypothesized that idiotype–anti-idiotype interactions with human lymphocytes contribute to their effects [27]. In 2005, Miri Blank and colleagues reviewed evidence on pIgG formulations and concluded that research into idiotype-specific IgG recognition could yield novel therapeutic approaches [28]. Such strategies might also inform the design of idiotype-based vaccines, with particular relevance for immunodeficient patients [29]. Unfortunately, technological limitations have restricted these investigations, which only became more feasible in the past decade and remain relatively underexplored.

Together, these findings provide the theoretical foundation for a series of in vitro studies that have assessed the direct effects of pIgG on murine and human lymphocytes, using donor pools stratified by immunological status (e.g., atopic or infectious diseases) [30–46]. This extensive body of work has demonstrated that variability in pIgG repertoires can functionally modulate diverse immune cell populations, including CD4⁺ T cells, CD8⁺ T cells, γδ T cells and B cells. These results support the hypothesis that circulating pIgG repertoires continuously interact with and regulate immune functions in both health and disease [47–49].

In summary, the current state of the literature highlights the clear immunomodulatory potential of pIgG but leaves unresolved fundamental questions regarding its specific interactions, direct effects, endogenous protein targets, and relationships with pathogen recognition. Addressing these aspects, as we propose in the present study, may substantially advance the therapeutic use of pIgG in human disease.

## Results

### pIgG differentially modulates CD4⁺ T, CD8⁺ T, γδ T, and B-cell phenotypes and cytokine profiles

**Fig. 1:**
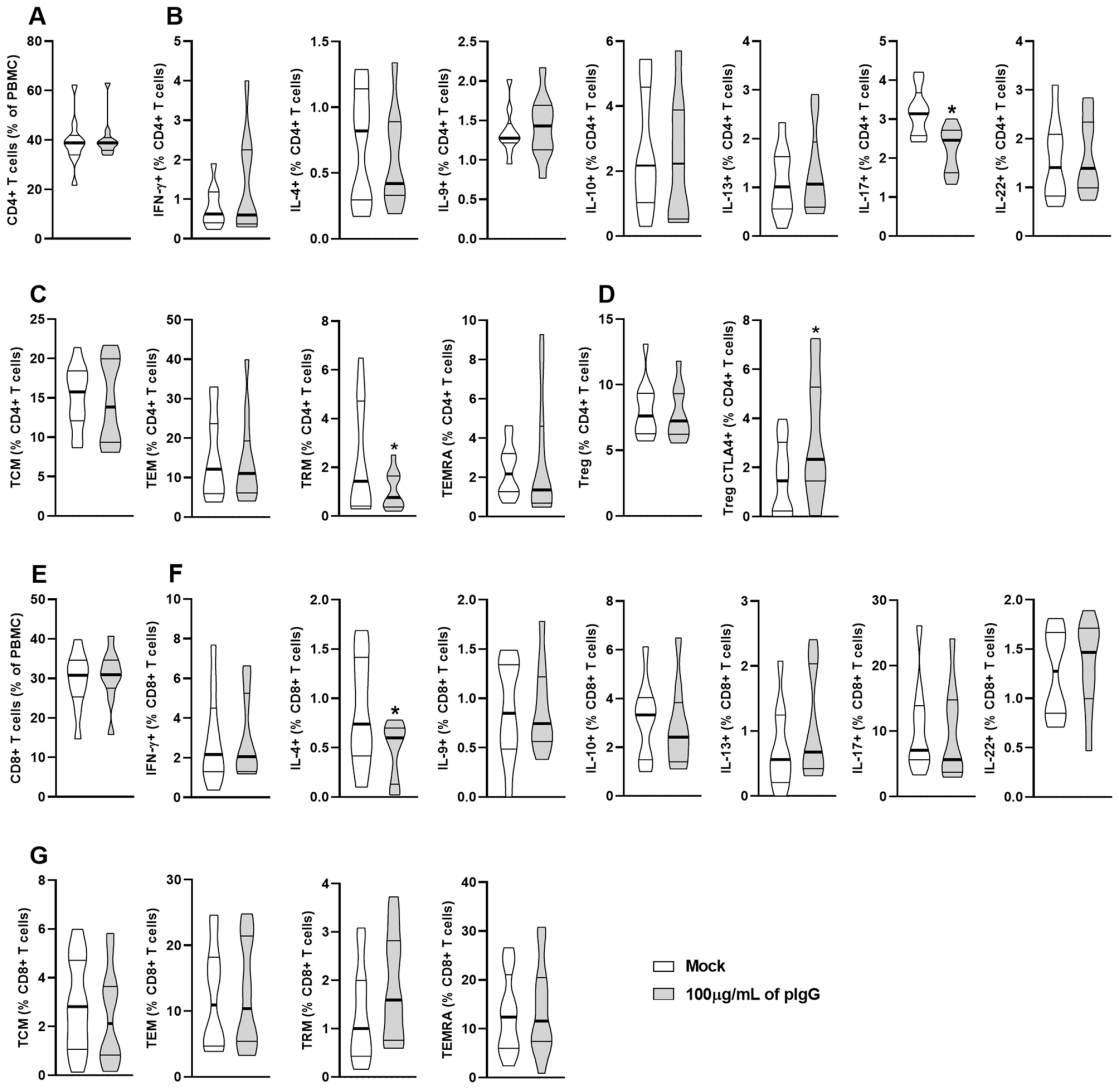
pIgG modulates CD4⁺ and CD8⁺ T-cell cytokine production and subset distribution. Peripheral blood mononuclear cells (PBMCs) from healthy donors were cultured for 3 days under mock conditions or with polyvalent IgG (pIgG; 100 µg/mL) and analyzed by flow cytometry. (**A**) Frequency of CD4⁺ T cells. (**B**) Intracellular cytokine production by CD4⁺ T cells. (**C**) Distribution of CD4⁺ T-cell subsets: central memory (TCM), effector memory (TEM), tissue-resident memory (TRM), and terminal effector memory RA (TEMRA). (**D**) Frequency of regulatory T cells (Treg) and CTLA-4 expression. (**E**) Frequency of CD8⁺ T cells. (**F**) Intracellular cytokine production by CD8⁺ T cells. (**G**) Distribution of CD8⁺ T-cell subsets (TCM, TEM, TRM, TEMRA). *p ≤ 0.05 vs. mock

After three days of culture, pIgG treatment did not affect the frequency of viable CD4⁺ T cells (Fig. 1A). Intracellular cytokine analysis revealed that pIgG significantly reduced the proportion of IL-17–producing CD4⁺ T cells, without altering IFN-γ, IL-4, IL-9, IL-10, IL-13, or IL-22 expression compared with mock-treated controls (Fig. 1B). In addition, pIgG decreased the frequency of tissue-resident memory (TRM) CD4⁺ T cells but did not modify the distribution of central memory (TCM), effector memory (TEM), or terminal effector memory RA (TEMRA) subsets (Fig. 1C). While the overall frequency of regulatory T cells (Tregs) remained unchanged, pIgG significantly increased CTLA-4 expression within this subset (Fig. 1D).

In CD8⁺ T cells, pIgG treatment likewise did not affect viability after three days of culture (Fig. 1E). Functional analyses showed that pIgG reduced the frequency of IL-4–producing CD8⁺ T cells, with no detectable changes in IFN-γ, IL-9, IL-10, IL-13, IL-17, or IL-22 production (Fig. 1F). The distribution of CD8⁺ T-cell memory subsets (TCM, TEM, TRM, and TEMRA) was also unaffected (Fig. 1G).

**Fig. 2:**
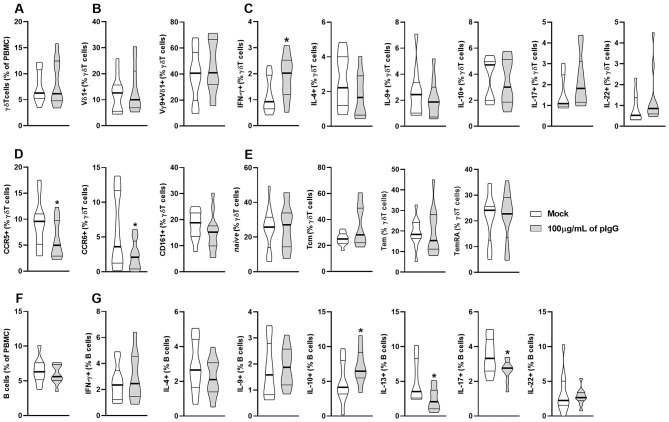
pIgG modulates γδ T-cell phenotype and B-cell cytokine production. PBMCs from healthy donors were cultured for 3 days under mock conditions or with pIgG (100 µg/mL) and analyzed by flow cytometry. (**A**) Frequency of γδ T cells. (**B**) Distribution of Vδ1⁺ and Vγ9⁺Vδ2⁺ subsets. (**C**) Intracellular cytokine production by γδ T cells. (**D**) Expression of CCR5, CCR6, and CD161 on γδ T cells. (**E**) Distribution of γδ T-cell differentiation subsets: naïve, TCM, TEM, and TEMRA. (**F**) Frequency of B cells. (**G**) Intracellular cytokine production by B cells. *p ≤ 0.05 vs. mock

Regarding γδ T cells, pIgG exposure did not influence viability (Fig. 2A) or the relative proportions of Vδ1⁺ and Vγ9Vδ2⁺ subsets (Fig. 2B). However, pIgG increased the frequency of IFN-γ–producing γδ T cells, while IL-4, IL-9, IL-10, IL-17, and IL-22 levels remained unchanged (Fig. 2C). Moreover, pIgG reduced CCR5 and CCR6 expression on γδ T cells without affecting CD161 expression (Fig. 2D). The distribution of naïve, TCM, TEM, and TEMRA subsets showed no significant differences (Fig. 2E).

In B cells, pIgG did not alter viability after three days of culture (Fig. 2F). Nevertheless, pIgG increased the proportion of IL-10–producing B cells while reducing IL-13– and IL-17–producing subsets, with no effect on IFN-γ, IL-4, IL-9, or IL-22 production (Fig. 2G).

### pIgG modulates PBMC transcriptomic and microRNA profiles with immunomodulatory implications

**Fig. 3:**
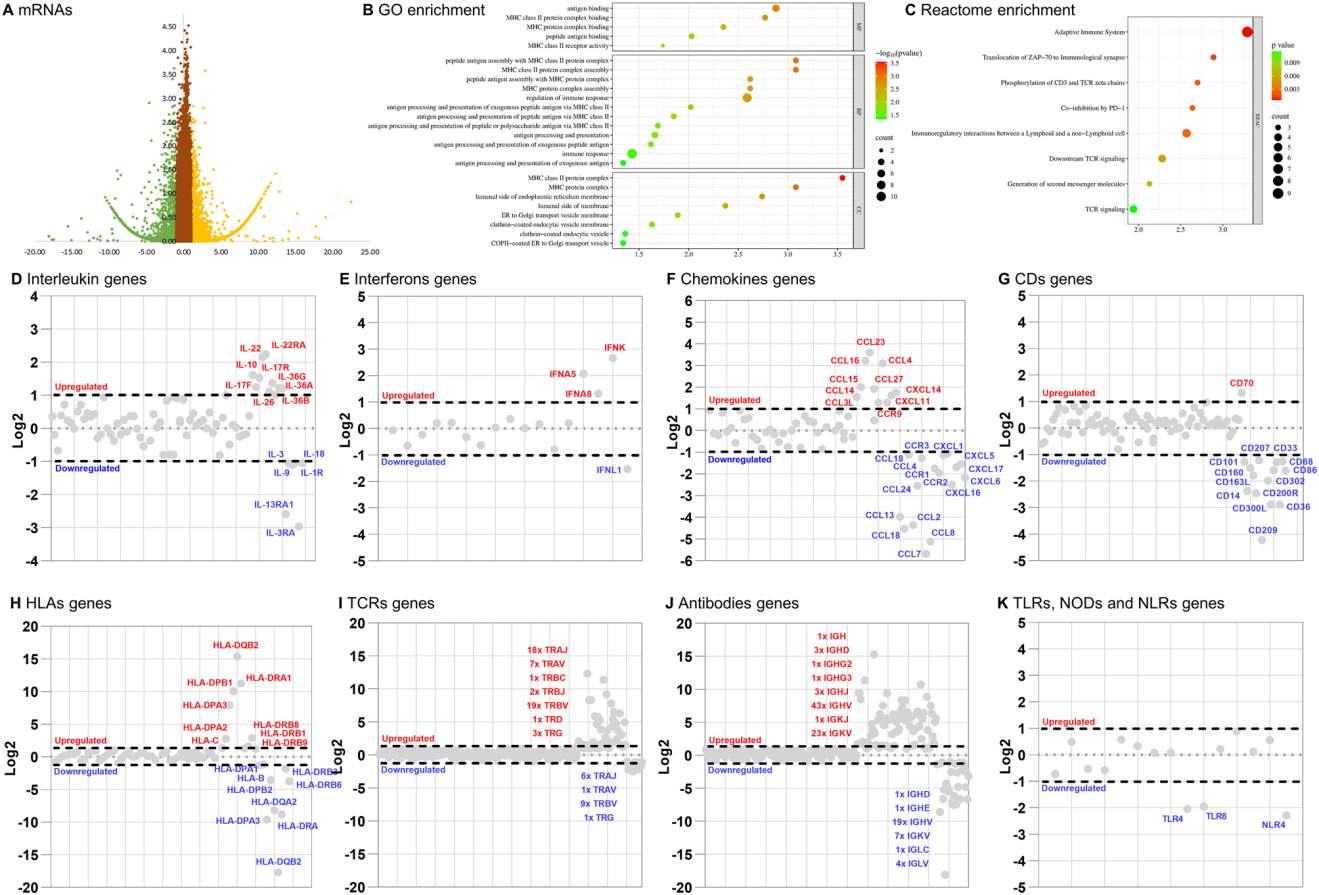
pIgG induces transcriptomic remodeling in PBMCs. PBMCs were cultured for 3 days under mock conditions or with pIgG (100 µg/mL), followed by RNA sequencing. (**A**) Differentially expressed genes. (**B**) Gene Ontology (GO) enrichment analysis. (**C**) Reactome pathway enrichment. Expression profiles of (**D**) interleukin-related genes, (**E**) interferon-related genes, (**F**) chemokines, (**G**) cluster of differentiation (CD) genes, (**H**) human leukocyte antigen (HLA) genes, (**I**) T-cell receptor (TCR) genes, (**J**) immunoglobulin genes, and (**K**) pattern-recognition receptor genes, including Toll-like receptors (TLRs), nucleotide-binding oligomerization domain (NOD) receptors, and NOD-like receptors (NLRs). Differential expression is shown using a log₂ fold-change threshold of ± 1

To explore potential transcriptional effects, we compared the mRNA expression profiles of PBMCs cultured under mock and pIgG-treated conditions. As shown in Fig. 3A, pIgG exposure resulted in the upregulation of 4,820 genes and the downregulation of 2,160 genes, while 25,173 genes remained unaltered. Gene Ontology (GO) enrichment analysis of the top 50 modulated genes revealed significant associations with pathways involved in MHC class II function and antigen processing (Fig. 3B). Similarly, Reactome enrichment indicated strong relevance for T-cell antigen recognition and activation pathways (Fig. 3C), suggesting that pIgG influences molecular networks central to adaptive immune regulation.

A more detailed analysis of selected immunologically relevant gene groups revealed that pIgG profoundly affected multiple cytokine and receptor families. Within the interleukin family, pIgG enhanced the expression of IL10, IL17F, IL17R, IL22, IL22R, IL26, and IL36A/B/G, while reducing the expression of IL1R, IL3, IL3R, IL9, IL13, and IL13R (Fig. 3D). Among interferon-related genes, pIgG upregulated IFNK and IFNA transcripts, whereas IFNL expression was decreased (Fig. 3E). The chemokine profile also showed extensive remodeling: upregulated genes included CCL3L, CCL4, CCR9, CXCL11, CXCL14, CCL14, CCL15, CCL16, and CCL23, while CXCL1, CCR1, CCL2, CCR3, CCL4, CXCL5, CXCL6, CCL7, CCL8, CCL13, CXCL16, CXCL17, CCL18, and CCL24 were downregulated (Fig. 3F).

Analysis of CD-molecule transcripts demonstrated selective modulation, with CD70 being the only upregulated marker, whereas CD14, CD33, CD36, CD68, CD86, CD101, CD160, CD163L, CD200R, CD207, CD209, CD300L, and CD302 were significantly downregulated (Fig. 3G). A comparable pattern of balanced regulation was observed among HLA genes: three HLA-DP, one HLA-DQ, four HLA-DR, and one HLA-C gene were upregulated, whereas three HLA-DP, one HLA-DQ, three HLA-DR, and one HLA-B gene were downregulated (Fig. 3H).

Rearrangement of genes associated with clonal receptors was also evident. Fifty-one T-cell receptor (TR) genes were upregulated and 17 were downregulated (Fig. 3I). In contrast, the immunoglobulin repertoire - comprising IGH, IGK, and IGL genes - displayed 78 upregulated and 33 downregulated transcripts (Fig. 3J). Finally, analysis of innate-immunity receptor families indicated that pIgG selectively reduced the expression of TLR4, TLR8, and NLR4, without detectable induction of other TLR, NOD, or NLR members (Fig. 3K).

**Fig. 4:**
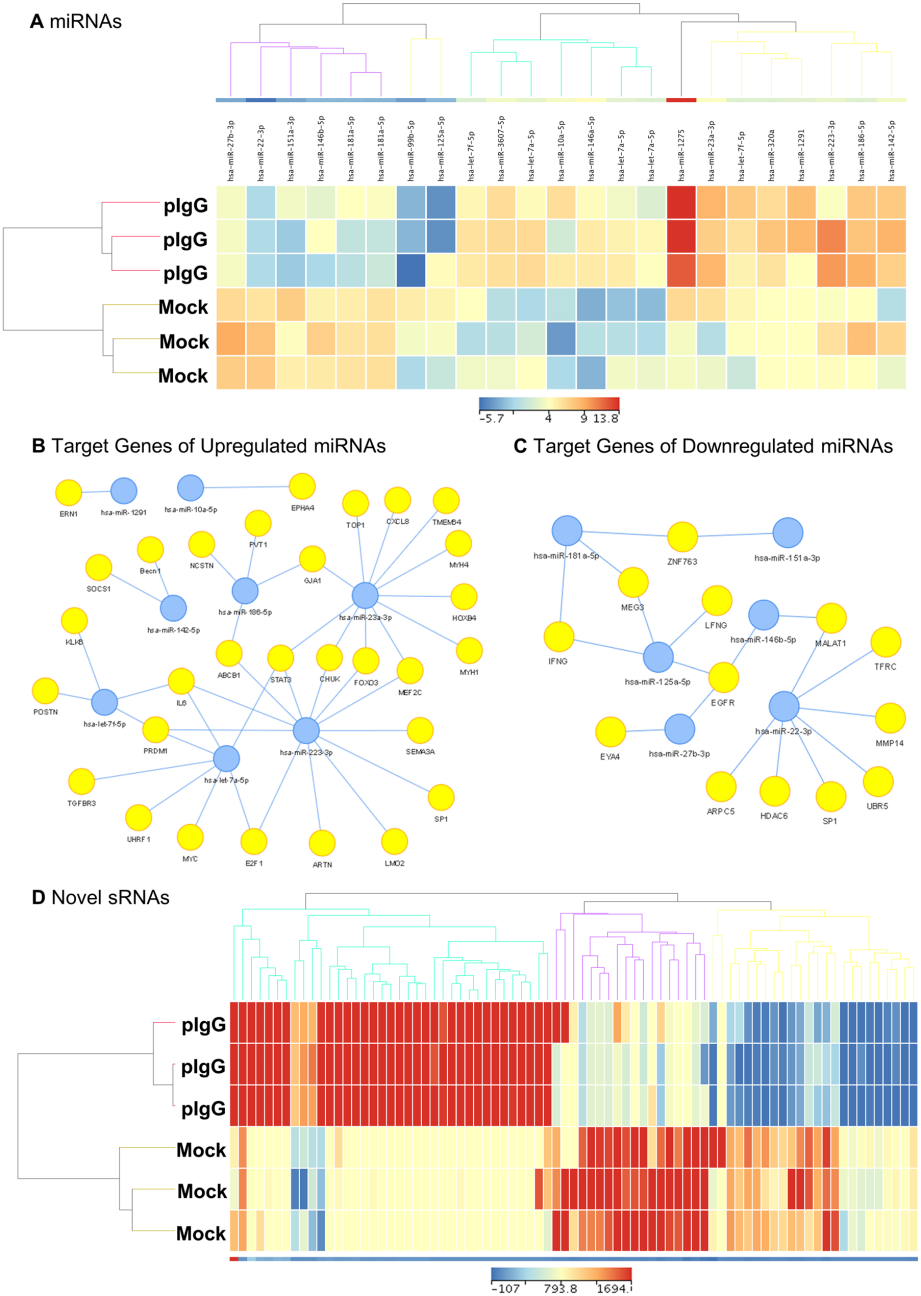
pIgG alters the small RNA profile of PBMCs. PBMCs were cultured for 3 days under mock conditions or with pIgG (100 µg/mL), followed by small RNA sequencing. (**A**) Differentially expressed microRNAs (miRNAs). (**B**) Predicted target genes of upregulated miRNAs. (**C**) Predicted target genes of downregulated miRNAs. (**D**) Differentially expressed novel small RNAs (sRNAs). Blue nodes represent miRNAs and yellow nodes represent predicted target genes

To assess potential epigenetic modulation, we next examined small RNA (sRNA) expression in PBMCs cultured under the same conditions. Nineteen microRNAs (miRNAs) were differentially expressed (Fig. 4A). Among these, a cluster comprising seven miRNAs - hsa-miR-27b-3p, hsa-miR-22-3p, hsa-miR-151a-3p, hsa-miR-146b-5p, hsa-miR-181a-5p, hsa-miR-99b-5p, and hsa-miR-125a-5p - was consistently downregulated in pIgG-treated cells. In contrast, three clusters encompassing eleven miRNAs - hsa-let-7f-5p, hsa-miR-3607-5p, hsa-let-7a-5p, hsa-miR-10a-5p, hsa-miR-1275, hsa-miR-23a-3p, hsa-miR-320a, hsa-miR-1291, hsa-miR-223-3p, hsa-miR-186-5p, and hsa-miR-142-5p - were upregulated. Collectively, these findings reveal that pIgG exposure induces broad transcriptional and post-transcriptional remodeling in PBMCs, engaging both cytokine-signaling networks and miRNA-mediated regulatory mechanisms with potential implications for immune modulation.

Predicted target analysis revealed that upregulated miRNAs were associated with genes involved in immune regulation, including SOCS1, IL6, STAT3, and CXCL8 (Fig. 4B), whereas downregulated miRNAs targeted key immune genes such as IFNG (Fig. 4C). Some target genes were shared across both up- and downregulated miRNAs, as shown in Supplementary Figure S1, which also displays the total number of predicted targets for each miRNA.

In parallel, 79 novel sRNAs were differentially expressed between conditions (Fig. 4D), including 37 upregulated and 42 downregulated clusters. Collectively, these findings demonstrate that pIgG induces distinct transcriptional and post-transcriptional signatures, suggesting that IgG repertoires may influence the epigenetic landscape of immune regulation.

### Direct interaction of pIgG with lymphocyte membranes independent of early apoptosis, and proteomic mapping of potential targets

**Fig. 5:**
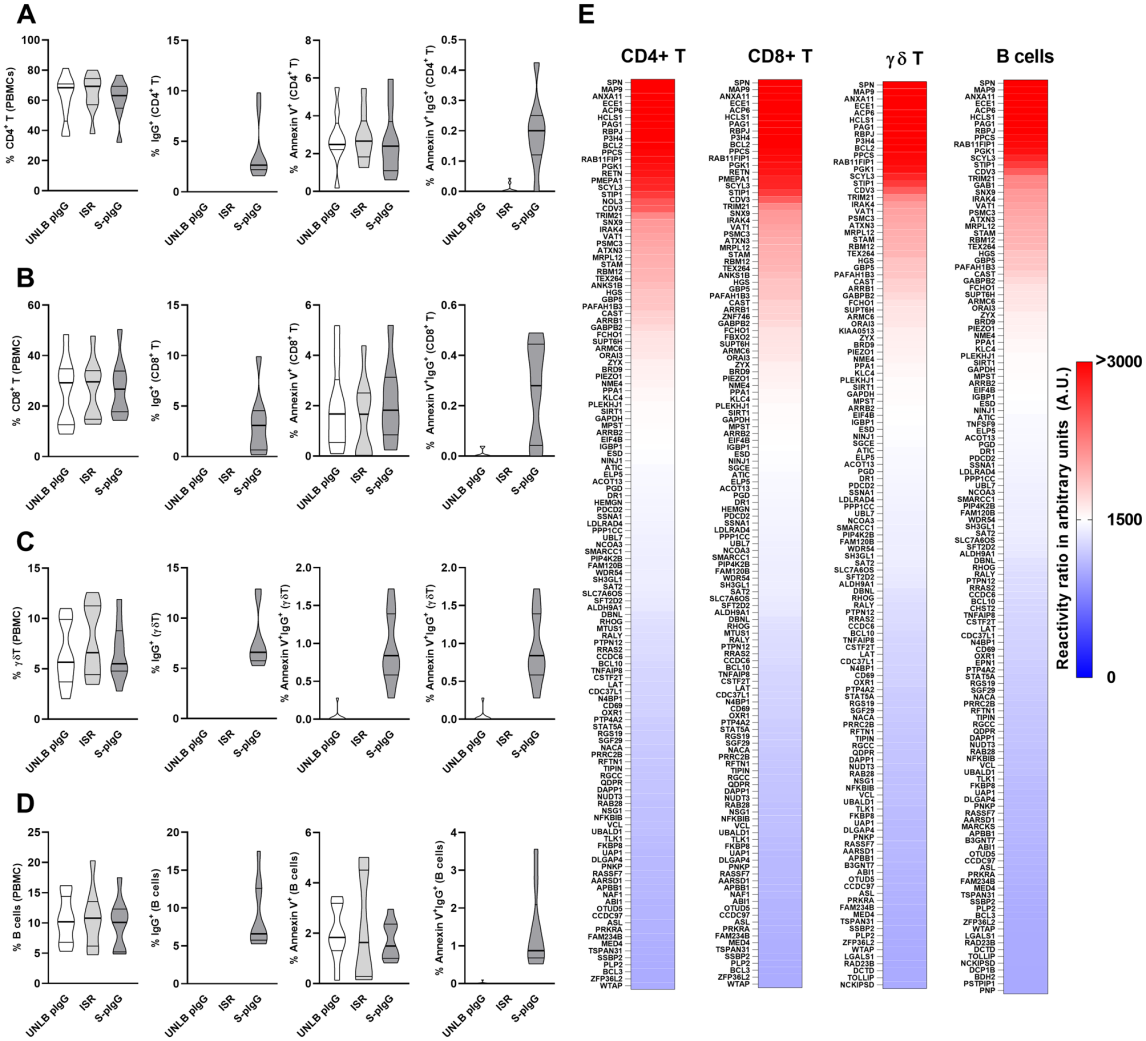
pIgG binds lymphocytes without inducing apoptosis and targets cell-expressed proteins. PBMCs were incubated for 30 min under mock conditions or with pIgG (100 µg/mL) and analyzed by flow cytometry. (A–D) IgG binding and Annexin V staining in (**A**) CD4⁺ T cells, (**B**) CD8⁺ T cells, (**C**) γδ T cells, and (**D**) B cells. (**E**) Heatmap summarizing proteins expressed by CD4⁺ T cells, CD8⁺ T cells, γδ T cells, and B cells that were targeted by pIgG according to the defined reactivity threshold

To determine whether pIgG directly interacts with lymphocyte membranes, specific staining was performed after 30 min of incubation. pIgG treatment did not alter the frequency of viable CD4⁺ T cells, and only ~ 3% displayed membrane-bound IgG (Fig. 5A). Early apoptosis rates remained low (median 2–3%), and double-positive IgG⁺/Annexin V⁺ cells accounted for only ~ 0.2% of the population, indicating that membrane binding was not associated with apoptosis. Similar results were obtained for CD8⁺ T cells (~ 3% binding), γδ T cells (~ 7% binding), and B cells (~ 6% binding), all showing low apoptosis and minimal IgG⁺/Annexin V⁺ overlap (Figs. 5B–D).

Since all lymphocyte subsets exhibited comparable membrane binding without apoptosis, we next mapped potential pIgG targets. Proteomic profiling identified 130 targeted proteins in CD4⁺ T cells, 132 in CD8⁺ T cells, 129 in γδ T cells, and 134 in B cells (Fig. 5E).

We extended this analysis to other peripheral immune cells. Among 9,494 monocyte proteins, 139 were recognized by pIgG; similarly, 134 of 9,048 myeloid dendritic cell proteins, 129 of 9,089 plasmacytoid dendritic cell proteins, 131 of 8,599 neutrophil proteins, 132 of 8,818 basophil proteins, 128 of 8,701 eosinophil proteins, 130 of 8,979 NK-cell proteins, and 124 of 9,036 MAIT-cell proteins showed strong reactivity (Supplementary Figure S2).

**Fig. 6:**
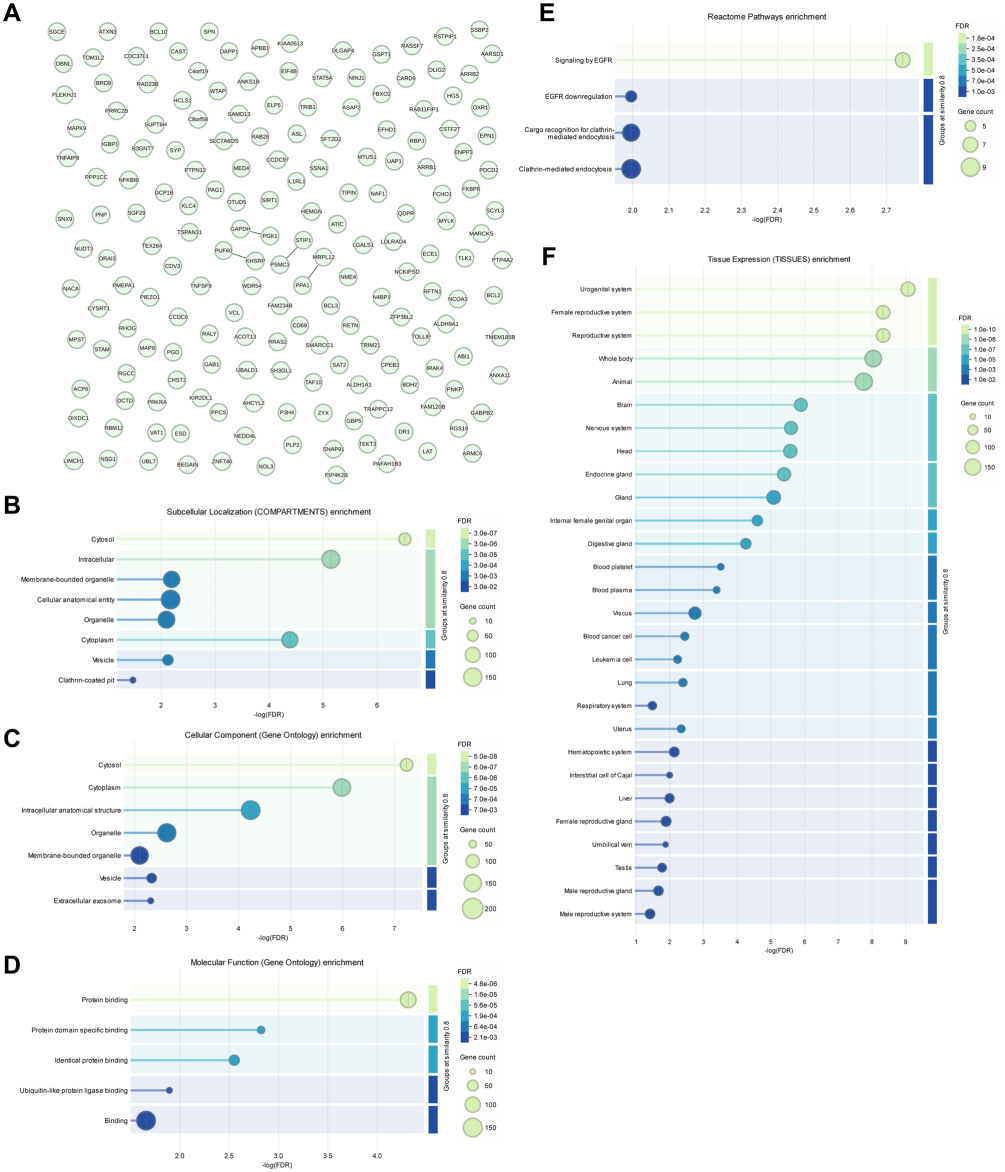
Protein–protein interaction network (PPIN) analysis of pIgG-targeted proteins. Proteins differentially targeted by pIgG were analyzed for functional enrichment. (**A**) Protein homology relationships. (**B**) Subcellular localization enrichment (COMPARTMENTS database). (**C**) Cellular component enrichment (Gene Ontology, GO). (**D**) Molecular function enrichment (GO). (**E**) Reactome pathway enrichment. (**F**) Tissue expression enrichment (TISSUES database). All enrichments are shown at false discovery rate (FDR) < 0.05

Integration of the proteins recognized in CD4⁺ T, CD8⁺ T, γδ T, and B cells yielded 180 unique targets. STRING network analysis revealed limited homology, with only four protein pairs displaying detectable interactions (Fig. 6A). Subcellular localization enrichment (COMPARTMENTS database) indicated associations with the cytosol, intracellular membrane-bounded organelles, cytoplasm, vesicles, and clathrin-coated pits (Fig. 6B). Consistent results were obtained by GO cellular-component analysis (Fig. 6C).

Molecular-function enrichment demonstrated overrepresentation of general protein binding, ubiquitin-like protein ligase binding, and related binding terms (Fig. 6D). Reactome pathway analysis highlighted enrichment in EGFR signaling and downregulation and clathrin-mediated endocytosis (Fig. 6E). Tissue-expression enrichment revealed significant associations with 28 tissues, including urogenital, reproductive, nervous, respiratory, hepatic, hematopoietic, neoplastic, and endocrine systems, indicating a broad and complex spectrum of pIgG-recognized proteins (Fig. 6F).

### Tracing the origins of pIgG recognition using human pathogen-derived epitopes

To further characterize the idiotypic repertoire of polyvalent IgG (pIgG), we assessed its reactivity toward pathogen-derived epitopes.

**Fig. 7:**
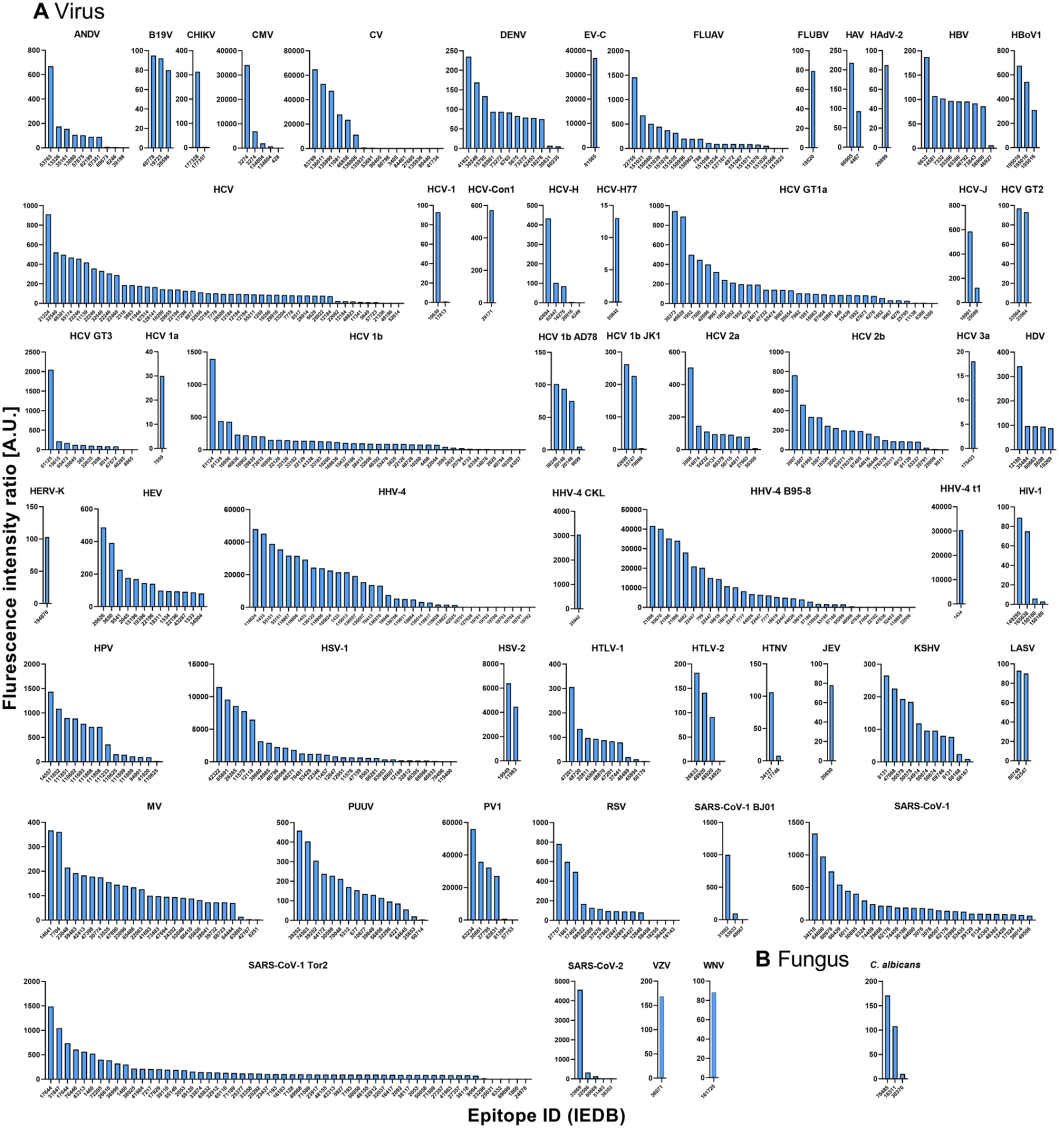
Reactivity of pIgG against viral and fungal pathogen-derived epitopes. Bar graphs illustrate the reactivity of polyvalent IgG (pIgG) against pathogen-derived linear epitopes identified by Immune Epitope Database (IEDB) IDs. The y-axis represents the fluorescence intensity ratio (arbitrary units – A.U.), reflecting relative epitope recognition intensity, and the x-axis indicates individual epitope IDs. (**A**) Viral epitopes. (**B**) Fungal epitopes. Only epitopes that met the predefined reactivity threshold are shown. A complete list of recognized epitopes and the full set of evaluated epitopes is provided in Supplementary Table S3

Analysis of viral epitopes revealed substantial reactivity across 56 evaluated viruses or viral strains (Fig. 7A), with hepatitis C virus (HCV) contributing the highest number of recognized epitopes. Notably, pIgG also recognized epitopes from pathogens excluded from blood donation criteria, confirming its extensive idiotypic diversity. Within the fungal panel, recognition was restricted to Candida albicans (Fig. 7B).

**Fig. 8:**
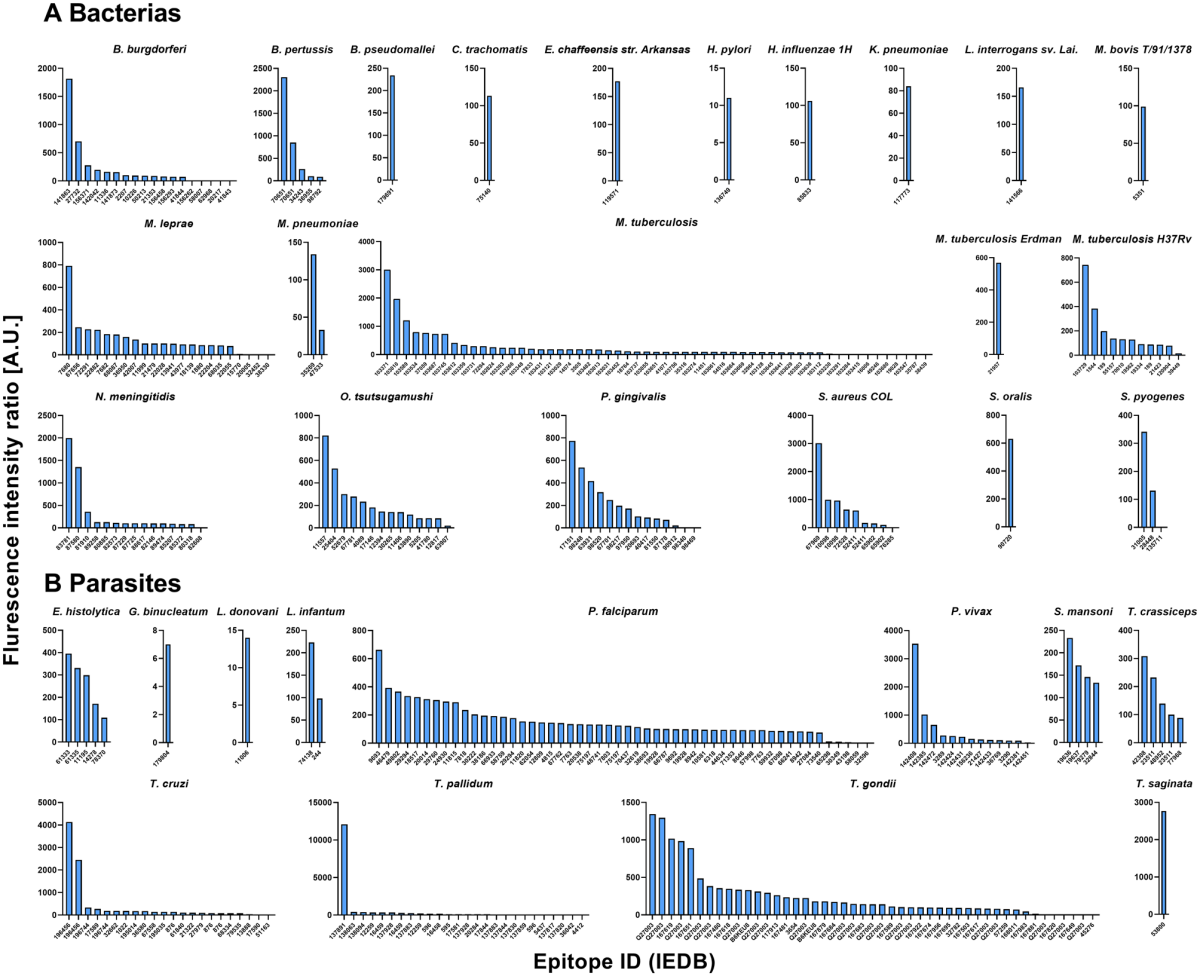
Reactivity of pIgG against bacterial and parasitic pathogen-derived epitopes. Bar graphs depict the reactivity of polyvalent IgG (pIgG) against pathogen-derived linear epitopes identified by Immune Epitope Database (IEDB) IDs. The y-axis represents the fluorescence intensity ratio (arbitrary units – A.U.), and the x-axis corresponds to individual epitope IDs. (**A**) Bacterial epitopes. (**B**) Parasitic epitopes. Only epitopes exceeding the defined reactivity threshold are displayed. Detailed lists of recognized epitopes and all evaluated epitopes are available in Supplementary Table S3

Among 53 bacterial species tested, epitopes from 21 were recognized by pIgG (Fig. 8A), with Mycobacterium tuberculosis exhibiting the highest frequency of recognized sequences. Within the parasitic panel, reactivity was detected toward epitopes from 12 of the 39 analyzed species, predominantly Plasmodium falciparum and P. vivax (Fig. 8B).

Epitope similarity analysis (Supplementary Figure S3) identified 947 recognized epitopes, corresponding to 942 unique sequences (full list of epitopes recognized by the pIgG and a full lista of all evaluated epitopes are provided in Supplementary Table S3, tabs 1 and 2). Clustering at an 80% sequence-identity cutoff yielded 686 distinct clusters, indicating that the extensive recognition breadth of pIgG arises from a genuinely diverse idiotypic repertoire rather than from sequence redundancy.

## Discussion

Our observation that pIgG suppresses IL-17 production by CD4⁺ T cells without broadly affecting IFN-γ, IL-4, or IL-10 secretion is consistent with previous reports showing that intravenous immunoglobulin (IVIG) downregulates Th17-driven inflammation [50, 51]. In line with these studies, the increased CTLA-4 expression observed in Tregs—without changes in their overall frequency—accords with evidence that IVIG enhances Treg suppressive function rather than expansion [52]. Together, these findings indicate that pIgG recapitulates core immunomodulatory mechanisms of IVIG by reinforcing peripheral tolerance through functional enhancement of regulatory circuits.

Beyond conventional αβ T cells, pIgG also influenced innate-like lymphocyte populations. The induction of IL-10–producing B cells parallels the emergence of regulatory B cells during IVIG therapy [53], while the increased frequency of IFN-γ–producing γδ T cells together with reduced CCR5 and CCR6 expression suggests a shift toward regulated migratory and effector programs. These findings extend the immunomodulatory effects of pIgG to innate-like lymphocytes, implicating them as contributors to IgG-mediated immune homeostasis. Although IVIG has been reported to modulate natural killer (NK) cell activation and cytotoxic function, NK-cell–specific phenotyping was beyond the scope of the present study and represents an important direction for future investigation.

Transcriptomic reprogramming induced by pIgG revealed enrichment of MHC-II and T-cell receptor (TCR) signaling pathways, consistent with broad immune recalibration. Coordinated induction of IL10 and IL22, together with repression of IL1R-, IL3R-, and IL13R-associated pathways, supports a shift from pro-inflammatory toward regulatory and tissue-protective signaling networks [54–56]. Accordingly, pIgG appears to suppress pathogenic inflammation while preserving physiological repair mechanisms, as further reflected by attenuation of Th2-associated IL-13 signaling and IL-1R/MyD88-dependent inflammatory pathways [57–59].

In parallel, pIgG reduced expression of innate pattern-recognition receptors, including TLR4, NLR4, and CD14, which are central to inflammatory amplification. Downregulation of TLR4 and CD14 is associated with reduced NF-κB activation and diminished production of pro-inflammatory cytokines such as IL-1β and TNF-α [60, 61], consistent with prior evidence that IgG immune complexes inhibit TLR-mediated signaling through interference with CD14/TLR4 interactions and induction of negative MyD88 regulators [62].

Beyond innate sensing, pIgG also modulated genes encoding co-stimulatory and inhibitory receptors, including CD101, CD160, and CD86. Upregulation of CD101 and CD160 is compatible with reinforcement of inhibitory signaling and restraint of excessive effector activation, whereas modulation of CD86 may bias antigen-presenting cell interactions toward CTLA-4–dependent inhibitory pathways rather than CD28-mediated costimulation [63–66]. Together, these transcriptional changes indicate coordinated recalibration of immune checkpoints that balance activation and suppression.

Another striking observation was the bidirectional regulation of genes encoding the T-cell receptor (TCR) and B-cell receptor (BCR) components. pIgG upregulated variable and constant gene segments of TCR chains—TRAJ, TRAV, TRBC, TRBJ, TRBV, TRD, and TRG—while selectively downregulating others (TRAJ, TRAV, TRBV, and TRG), indicating a reconfiguration of receptor diversity. Such transcriptional reshaping may reflect idiotype-driven feedback within the adaptive compartment, where IgG idiotypes can engage BCR and TCR variable regions through internal image recognition [67–69]. Previous studies demonstrated that IVIG can modulate TCR signaling thresholds and promote anergy or tolerance via FcγRIIb and associated phosphatases [70]. The heterogeneous TCR transcript changes observed here could represent a molecular signature of this idiotypic regulation, potentially influencing antigen sensitivity and repertoire composition.

Similarly, differential regulation of BCR-related genes—including upregulation of IGH, IGHD, IGHG2, IGHG3, IGHJ, IGHV, IGKJ, and IGKV, alongside downregulation of IGHD, IGHE, IGHV, IGKV, IGLC, and IGLV—suggests that pIgG induces selective remodeling of B-cell receptor expression. IVIG has been shown to interact directly with BCR and FcγR pathways, promoting the emergence of IL-10–producing Bregs while repressing autoreactive clones [71, 72];. The parallel upregulation of IgG subclasses (IGHG2, IGHG3) and certain variable region genes may indicate compensatory diversification under homeostatic control, aligning with idiotypic network theory, wherein antibody repertoires self-regulate through stimulatory and inhibitory loops [67, 73–75]. Thus, pIgG appears to modulate adaptive receptor gene expression in a manner consistent with systemic idiotypic feedback.

The differential regulation of interferons observed here - upregulation of IFN-α and IFN-κ with concomitant suppression of IFN-λ - also aligns with a controlled activation of antiviral and regulatory programs. Type I interferons can recalibrate adaptive responses, promoting Treg stability and tolerance under steady-state conditions [76, 77]. In contrast, type III interferons (IFN-λ) primarily act at mucosal barriers, and their downregulation may serve to prevent excessive epithelial activation. These results suggest that pIgG orchestrates a transcriptional balance reminiscent of the transient “reset” induced by therapeutic IVIG, characterized by tonic IFN-I activity and broad chemokine suppression.

At the post-transcriptional level, the altered miRNA repertoire provides an additional regulatory layer. Upregulation of let-7 family members and miR-23a has been associated with attenuation of Th17 polarization and reduced inflammatory trafficking [78, 79]. Conversely, miR-223-3p, known to suppress NLRP3-inflammasome activation [80] and dendritic-cell antigen presentation [81], was also increased, suggesting that pIgG curtails innate inflammatory amplification loops. The simultaneous downregulation of miR-181a-5p, a key regulator of TCR signaling thresholds [82], may contribute to the observed upregulation of CTLA-4 in Tregs, consistent with data showing that miR-181a/b-1 deletion enhances Treg suppressive capacity [83]. Together, these findings support a model in which pIgG is associated with coordinated epigenetic and transcriptional remodeling of immune cells, potentially contributing to the maintenance of regulatory balance through miRNA-linked modulation of activation thresholds.

The concordance between small-RNA and mRNA profiles suggests that pIgG exposure is associated with coordinated signaling and gene-regulatory remodeling. Although direct membrane binding of pIgG did not affect lymphocyte viability, these interactions are compatible with non-canonical modes of antibody recognition and intracellular signaling. In this context, the cytosolic Fc receptor TRIM21 represents a biologically plausible mediator, as it binds internalized IgG and can link antibody recognition to proteasomal processing and downstream signaling events [84]. TRIM21 activity is tightly regulated by phosphorylation and autoinhibitory domains [85], providing a mechanistic framework through which extracellular antibody engagement could, in principle, interface with intracellular immune sensing and transcriptional responses.

Consistent with this conceptual framework, proteomic profiling identified approximately 130 pIgG-recognized proteins per lymphocyte subset and 180 shared targets enriched in cytosolic and vesicular compartments, as well as in ubiquitin-ligase binding and clathrin-mediated endocytosis pathways. These patterns are compatible with intracellular processing routes described for antibody–antigen complexes, including ubiquitin–proteasome–associated mechanisms [86]. In addition, enrichment of EGFR-signaling components suggests that pIgG interactions may intersect receptor-trafficking networks. Nevertheless, the involvement of TRIM21 or related intracellular antibody-sensing pathways in the present system remains hypothetical and will require direct experimental validation.

Because transcriptomic analyses were performed on pooled donor samples, gene-level statistical inference is inherently limited. Nevertheless, the concordance observed across transcriptomic signatures, cellular phenotypes, and proteomic target profiles supports the biological relevance of the identified regulatory patterns. Likewise, miRNA–target associations were inferred using established in silico and experimentally curated databases and should therefore be interpreted as supportive and hypothesis-generating, rather than as evidence of direct mechanistic regulation.

Future studies should focus on experimental validation of selected miRNAs identified in this work. Such approaches may include targeted gain- or loss-of-function strategies, reporter-based assays to confirm specific miRNA–mRNA interactions, and protein-level analyses to assess downstream effects on immune-regulatory signaling pathways. Validation in independent donor samples and in defined immune cell subsets will be particularly important to delineate cell-type–specific regulatory circuits and to clarify the contribution of miRNA-mediated mechanisms to the immunomodulatory effects of polyvalent IgG. Together, these efforts will help transition from systems-level associations toward a more detailed mechanistic understanding of IgG-driven regulatory networks.

The broad tissue enrichment of pIgG-recognized proteins - spanning hematopoietic, neural, endocrine, and epithelial systems - indicates that idiotypic interactions mediated by IgG extend far beyond the immune compartment. Classical idiotypic network theory, pioneered by Jerne [67] and later refined [47, 68, 69], envisioned antibodies as dynamic regulators of one another within a self-referential network. The present data broaden this framework, revealing idiotypic connectivity that transcends immune receptor networks to influence systemic immunophysiological regulation.

The extensive recognition of pathogen-derived epitopes, including viral (HCV), bacterial (*Mycobacterium*), fungal (*Candida*), and parasitic (*Plasmodium*) sequences, illustrates the remarkable diversity of the natural IgG repertoire. Because pIgG is derived from plasma donors who are seronegative for these pathogens, a mandatory criterion for blood donation, this reactivity is unlikely to reflect classical antigen-specific memory. Instead, it more plausibly arises from cross-reactive, autoreactive, natural, or anti-idiotypic antibody specificities that collectively contribute to the breadth of the IgG repertoire. Consistent with this interpretation, the high proportion of unique epitopes (942 of 948) and their clustering into 686 nonredundant groups argue against sequence redundancy or nonspecific binding and support the existence of genuine binding diversity within polyclonal IgG preparations. Together, these findings provide empirical support for the classical concept that polyclonal IgG continuously modulates immune tone through widespread, low-affinity interactions embedded within an idiotypic network.

In summary, our findings portray polyvalent IgG as a multilayered immunoregulatory agent that operates at the intersection of molecular recognition, signaling, and gene regulation. Through non-cytotoxic membrane binding- potentially sensed by intracellular receptors such as TRIM21- pIgG triggers post-transcriptional and transcriptional reprogramming that collectively attenuates pro-inflammatory Th17 activity, enhances regulatory (CTLA-4⁺ Treg and IL-10⁺ Breg) circuits, and modulates innate-like γδ T-cell function. The system-wide scope of pIgG interactions and its extensive idiotypic diversity suggest that IgG networks integrate immune regulation across cellular and tissue boundaries.

The “hooks without bait” theory [48], proposes that immunoglobulin molecules can modulate immune cell function independently of classical antigen engagement. In this model, the variable regions of IgG—shaped by prior immune history and idiotypic interactions—act as functional “hooks” capable of engaging immune receptors, intracellular sensors, or regulatory pathways even in the absence of their original cognate antigen (“bait”). Rather than mediating antigen-specific immunity, these interactions contribute to tonic immune regulation by shaping activation thresholds, cytokine profiles, and cellular differentiation programs. In the context of polyvalent IgG, which represents a highly diverse and pooled antibody repertoire, such antigen-independent interactions provide a conceptual framework for understanding how IgG can exert broad immunomodulatory effects across multiple immune cell types.

Together, the transcriptomic, miRNA, and proteomic data presented here substantiate this model, establishing pIgG as a dynamic orchestrator of immune equilibrium and a potential prototype for harnessing antibody networks in therapeutic immunoregulation.

## Materials and methods

### Samples

Peripheral blood mononuclear cells (PBMCs) were obtained from 20 healthy donors. Inclusion criteria required no history of allergic or atopic disease, no autoimmune disorders, and no use of medications known to influence lymphocyte activation or function.

### Characterization of polyvalent IgG (pIgG)

The purity of pIgG preparations was confirmed to exceed 95%, as previously validated by our group. Protein concentrations were determined using the Coomassie Protein Assay (Pierce, USA), and the distribution of IgG subclasses (IgG1–IgG4) was quantified with the Human IgG Subclass ELISA Kit (Thermo Fisher Scientific, USA) according to the manufacturer’s instructions. The subclass distribution is presented in Supplementary Figure S4.

Endotoxin levels were assessed with the Pierce LAL Chromogenic Endotoxin Quantitation Kit (Thermo Fisher Scientific, USA), confirming absence of contamination. For membrane-binding assays, pIgG was fluorescently labeled using the Zenon Human IgG Labeling Kit (Invitrogen, USA), which selectively blocks Fc domains to ensure specific Fab-mediated staining. PBMCs were incubated for 30 min with Zenon-labeled pIgG, or with labeling reagents alone as controls. The optimal IgG concentration (100 µg/mL) was empirically established in culture assays as previously described [46, 87]. Specificity was verified by pre-incubating samples with unlabeled IgG, which abolished subsequent Zenon staining. In parallel, early apoptosis was assessed using Annexin V staining with the Annexin V Apoptosis Detection Kit (Thermo Fisher Scientific, USA). Representative gating strategies for identification of IgG⁺ cells are provided in Supplementary Figure S5.

### Cell culture and flow cytometry

Cell culture procedures were performed as described previously (44, 45), using either pIgG (Endobulin/Kiovig; Baxter, Lessines, Belgium) or mock conditions without IgG. PBMCs (2 × 10⁶ cells per well) were seeded into 48-well plates (CoStar, USA) containing RPMI 1640 medium supplemented with 10% fetal bovine serum (FBS; HyClone III, USA) at a final volume of 400 µL. Cells were cultured for 72 h in the presence or absence of 100 µg/mL of the indicated IgG preparations.

Phenotypic characterization of T cells (CD4⁺, CD8⁺, regulatory T cells [Treg], and γδ T cells) and B cells was performed using fluorochrome-conjugated antibodies against CD3, CD4, CD8, CD19, CD25, CD45RA, CD45RO, CD27, CD69, CD127, CTLA-4, γδTCR, Vδ1TCR, Vδ2TCR, Vγ9TCR, CD161, CCR5, CCR6, and CCR7, together with appropriate isotype and fluorescence-minus-one (FMO) controls.

Gating was performed sequentially as follows: singlets → viable cells (Live/Dead Fixable Dead Cell Stain; Thermo Fisher Scientific, USA) → lymphocytes based on forward- and side-scatter properties (FSC/SSC). CD4⁺ T cells were defined as CD3⁺CD4⁺CD8⁻, CD8⁺ T cells as CD3⁺CD4⁻CD8⁺, B cells as CD19⁺, and γδ T cells as CD3⁺γδTCR⁺.

αβ T-cell differentiation subsets (CD4⁺ and CD8⁺) were defined based on CD45RA and CD27 expression as naïve (CD45RA⁺CD27⁺), central memory (TCM; CD45RA⁻CD27⁺), effector memory (TEM; CD45RA⁻CD27⁻), and terminal effector memory RA (TEMRA; CD45RA⁺CD27⁻). In parallel, memory and tissue-residency phenotypes were further characterized using CD45RO, CCR7, and CD69 expression to identify tissue-resident memory (TRM; CD45RO⁺CCR7⁻CD69⁺) subsets.

Regulatory T cells were identified within the CD4⁺ T-cell compartment as CD25⁺CD127⁻ cells, and CTLA-4 expression was evaluated within this population. Representative gating strategies for CD4⁺ and CD8⁺ T-cell subsets are provided in Supplementary Figure S6.

For γδ T cells, expression of CCR5, CCR6, and CD161 was assessed, and major subsets were defined as Vδ1⁺ (CD3⁺γδTCR⁺Vδ1⁺Vδ2⁻) and Vγ9⁺Vδ2⁺ (CD3⁺γδTCR⁺Vγ9⁺Vδ2⁺). Intracellular cytokine staining was performed by culturing PBMCs under identical conditions with Brefeldin A (Sigma, Israel) added during the final 6 h of culture. Following surface staining, cells were permeabilized with saponin and stained for IFN-γ, IL-4, IL-9, IL-10, IL-13, IL-17 A, and IL-22.

Antibody titrations were performed to determine optimal staining concentrations (1 µg/test). Cell viability and gating accuracy were verified using Live/Dead staining, isotype controls, and FMO controls.

Samples were acquired on an LSR Fortessa flow cytometer (BD Biosciences, USA), recording 100,000 lymphocyte-gated events per sample. UltraComp eBeads (Thermo Fisher Scientific, USA) were used for compensation. Data were analyzed in FlowJo software (Tree Star, USA).

### Human peripheral cell protein microarray

IgG recognition of human proteins was evaluated with HuProt™ Human Proteome Microarrays v4.0 (CDI Labs, Puerto Rico). The arrays contained 9,526, 9,505, 9,047, and 9,171 proteins annotated as expressed in CD4⁺ T, CD8⁺ T, γδ T, and B cells, respectively. Additional analyses included 9,494 proteins expressed by monocytes, 9,048 by myeloid dendritic cells (DCs), 9,089 by plasmacytoid DCs, 8,599 by neutrophils, 8,818 by basophils, 8,701 by eosinophils, 8,979 by natural killer (NK) cells, and 9,036 by mucosal-associated invariant T (MAIT) cells. Protein annotations were obtained from the Human Protein Atlas (full list for each cell population provided in Supplementary Table S1). All proteins were expressed in yeast as GST fusion proteins.

Each protein was spotted in duplicate across 20 blocks, together with alignment markers (Rhodamine + IgG647) and internal controls. Slides were washed three times with PBST (PBS containing 0.05% Tween 20) for 10 s and blocked for 30 min with Rockland Blocking Buffer (MB-070). Arrays were then incubated with purified polyvalent IgG (pIgG; equivalent to a 1:500 dilution in PBST supplemented with 10% blocking buffer) for 16 h at 4 °C under orbital shaking (140 rpm). Detection was performed using DyLight 680–labeled goat anti-human IgG (Fc-specific; 0.1 µg/mL) for 45 min at room temperature. Arrays were scanned using an InnoScan 710-IR scanner (10 μm resolution, 680 nm laser, gain 30, low power).

Background was assessed with secondary antibody alone. TIFF images (16-bit grayscale) were processed in Mapix 9.1.0 (Innopsys). Median foreground and background intensities were calculated, duplicates averaged, and background-subtracted. Signals with ratios ≤ 2 were excluded, and a minimum background of 50 fluorescence units was applied to prevent inflated ratios.

### Identification of IgG targets and bioinformatics analysis

Proteins were considered candidate IgG targets when signal intensities exceeded mean + 3 SD of the pIgG group, as recommended (39, 40). Protein lists were analyzed in STRING (STRING Consortium, 2024) to generate protein–protein interaction networks (PPINs). Functional annotations included structural homology, co-expression, Reactome pathways, Gene Ontology (GO) biological processes and molecular functions, subcellular localization (COMPARTMENTS), and WikiPathways enrichment.

### Infectious disease epitope microarray

IgG epitope profiling was performed using the PEPperCHIP^®^ Infectious Disease Epitope Microarray (PEPperPRINT GmbH, Heidelberg, Germany), which comprises 4,344 linear peptides derived from 133 viral (including sub-strains), 53 bacterial (including sub-strains), and 39 parasitic (including multiple species) antigens, as well as one fungal antigen. The complete list of pathogens is provided in Supplementary Table S2.

Samples were incubated on slides, and IgG binding was detected with fluorescent secondary antibody. Hemagglutinin (HA) peptides were included as internal controls.

Signal intensities were extracted from 16-bit TIFF images. Duplicate spot intensities were averaged, and deviations > 40% were excluded unless validated manually. Spots with coefficients of variation (CV) > 30% were excluded unless deemed reliable. Corrected intensities were ranked to identify the most reactive epitopes. Thresholds were defined as mean HC intensity + 3 SD plus a signal ratio > 2 relative to the comparison group. Epitope clustering was conducted using the IEDB Analysis Resource (http://tools.iedb.org/main/) with an identity cutoff of 80% and no restrictions on peptide length.

### RNA extraction, small RNA and RNA-seq library preparation, and sequencing

Peripheral blood mononuclear cells (PBMCs) obtained from 20 healthy donors were pooled in equal proportions to generate representative samples for each experimental condition (mock-treated and pIgG-treated). Total RNA was extracted using the RNeasy Mini Kit (Qiagen GmbH) combined with TRIzol^®^ reagent (Thermo Fisher Scientific, Inc.), while small RNA (sRNA) fractions were isolated using the miRNeasy Mini Kit (Qiagen GmbH), following previously established protocols [88].

The concentration and integrity of total and small RNAs were assessed using a Qubit 2.0 Fluorometer with the miRNA HS Assay Kit (Thermo Fisher Scientific, Inc.) and a 4200 TapeStation system (Agilent Technologies, Santa Clara, CA, USA), respectively.

### Small RNA library preparation and sequencing

Small RNA libraries were generated for each experimental group using the Small RNA v1.5 Sample Preparation Kit (Illumina, San Diego, CA, USA) according to the manufacturer’s instructions. Sequencing was performed using Illumina’s standard small RNA workflow to produce single-end reads.

### Messenger RNA library preparation and sequencing

For transcriptome profiling, 100 ng of total RNA per condition was used to construct mRNA libraries with the TruSeq Stranded mRNA Library Prep Kit (Illumina, San Diego, CA, USA), which enriches for polyadenylated transcripts through oligo(dT) magnetic bead capture. Fragmented double-stranded cDNA was synthesized following the manufacturer’s protocol.

A pooled library (1,450 pg/µL), comprising samples from all experimental conditions, was sequenced at the CELA Core Facility, University of São Paulo. Sequencing was carried out on an Illumina NextSeq 500 platform to generate paired-end reads (2 × 75 bp). FASTQ files were produced using the bcl2fastq software (Illumina).

### Data processing and exploratory differential expression analysis

Raw sequencing reads were subjected to adapter trimming and quality filtering using Strand NGS (Strand Life Sciences, Bangalore, India). High-quality reads were aligned to the human reference genome (GRCh38/hg38), and gene and small RNA expression levels were quantified within the same platform. For mRNA analyses, expression values were normalized as reads per kilobase per million mapped reads (RPKM), whereas small RNA expression was quantified as read counts. RPKM normalization was applied to enable exploratory, within-sample expression scaling and fold-change–based comparisons in pooled samples and was not intended for replicate-based statistical inference.

Exploratory differential expression analysis was conducted using the DESeq2 algorithm implemented in Strand NGS. As only one representative sample per group was analyzed, adjusted p-values were not computed. Genes and small RNAs exhibiting log₂ fold change > 1 were considered upregulated, whereas those with log₂ fold change < − 1 were considered downregulated relative to mock-treated controls. RNA-seq and small RNA-seq analyses were performed as exploratory, hypothesis-generating approaches intended to integrate with functional and proteomic datasets, rather than as standalone inferential analyses.

### Functional and target interaction analyses

Functional over-representation analysis of the top upregulated and downregulated genes was performed using Gene Ontology (GO) terms and Kyoto Encyclopedia of Genes and Genomes (KEGG) pathways through the clusterProfiler R package [89]. An FDR-adjusted p-value (q-value) < 0.05 was considered statistically significant.

Predicted target genes of differentially expressed miRNAs identified in mock- and pIgG-treated groups were retrieved using the miRWalk 3.0 algorithm. Target genes for each miRNA were subsequently analyzed for GO enrichment and KEGG pathway classification. miRWalk v3 was further employed to identify significantly deregulated miRNA target genes in Reactome pathways, applying the Benjamini–Hochberg correction to control the false discovery rate (FDR) at *p* < 0.05. The miRWalk network algorithm was also used to explore potential interactions between differentially expressed miRNAs and their predicted mRNA targets.

### Targetome analysis

miRNA–target enrichment analysis was performed using the MIENTURNET platform (http://userver.bio.uniroma1.it/apps/mienturnet/). Target prediction was based on the miRTarBase database, applying a threshold of at least one validated interaction. The false discovery rate (FDR) threshold was set to 1 or < 0.05, depending on the analysis. Only targets supported by strong experimental evidence were included. All analyses were conducted in an unsupervised manner, and the resulting figures were automatically generated by the MIENTURNET platform.

## Electronic Supplementary Material

Below is the link to the electronic supplementary material.

Supplementary Material 1

Supplementary Material 2

Supplementary Material 3

Supplementary Material 4

## Data Availability

The full datasets used to produce the current study are available from the corresponding author upon reasonable request.
