# Supplementary material for: System-wide immunoregulation by polyvalent IgG: integrating transcriptomic, miRNA, and proteomic landscapes
Source: J Transl Med. 2026 May 30;24:983. doi: 10.1186/s12967-026-08289-6 (PMC13430737; doi:10.1186/s12967-026-08289-6)
Supplement: Supplementary file 1 — Supplementary Material 1 [file 12967_2026_8289_MOESM1_ESM.pdf]

Supplementary figure S1

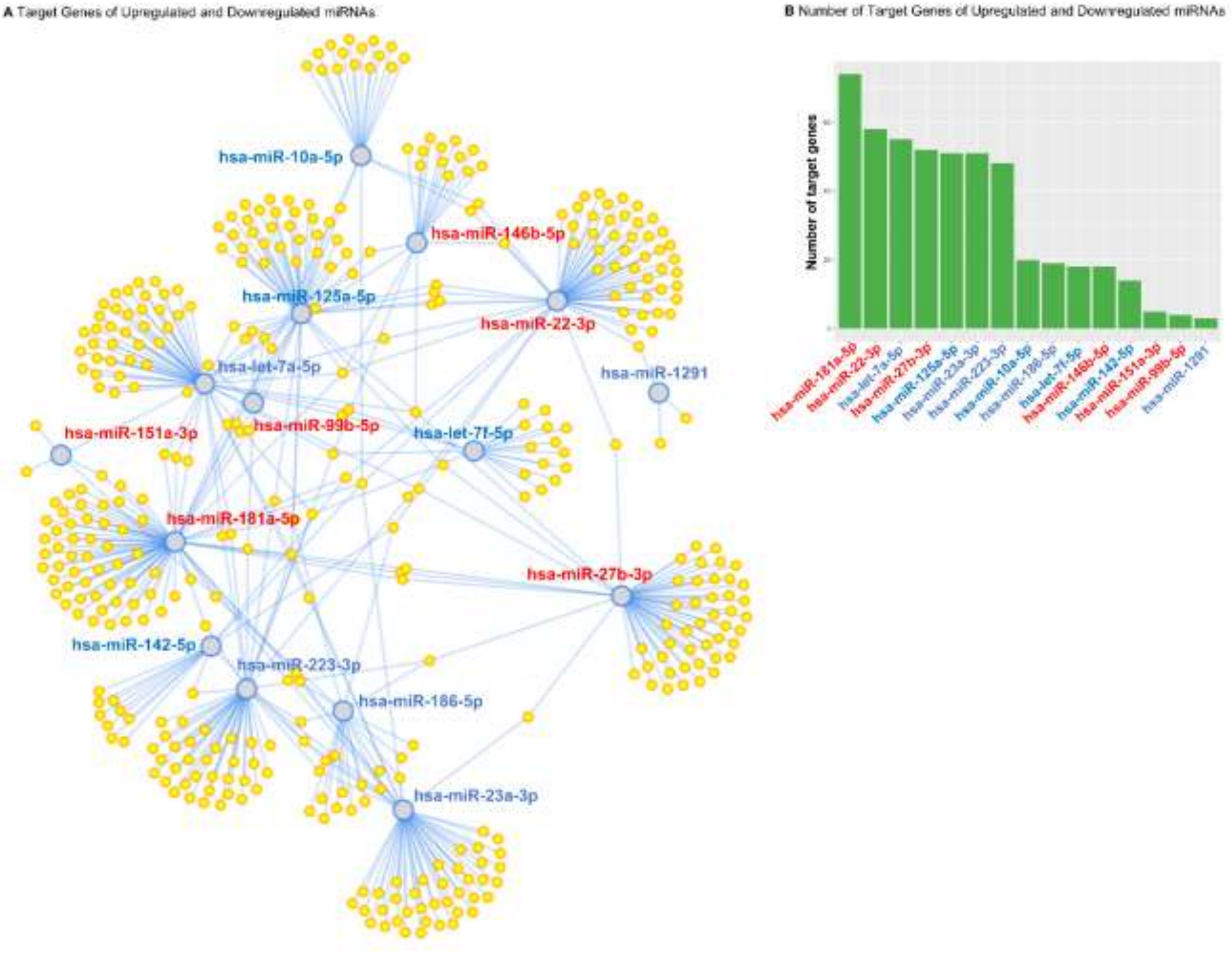

nalyzed,
with no
ı of 686

Supplementary Figure S1. Target gene interactions and counts associated with pIgG-modulated miRNAs. (A) Network visualization of target genes shared among miRNAs upregulated (red) and downregulated (blue) following pIgG treatment. (B) Number of predicted target genes corresponding to each upregulated (red) and downregulated (blue) miRNA modulated by pIgG.

Supplementary figure S2

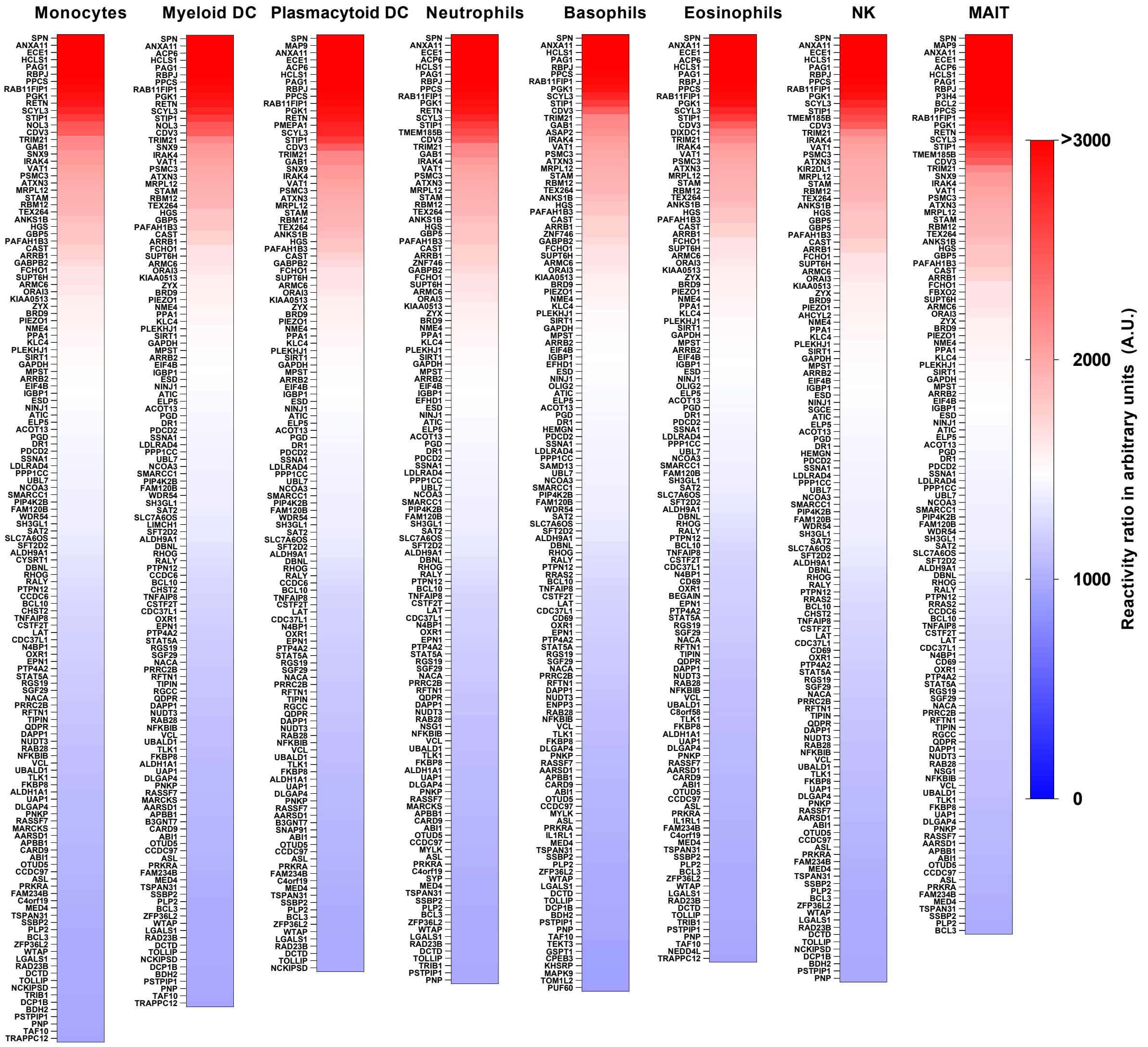

Supplementary Figure S2. Targeting of peripheral lymphocyte proteins by pIgG. Proteome-wide profiling of pIgG reactivity was performed using human protein microarrays. The heatmaps display proteins targeted across various immune cell subsets, including monocytes, myeloid dendritic cells, plasmacytoid dendritic cells, neutrophils, basophils, eosinophils, natural killer (NK) cells, and mucosal-associated invariant T (MAIT) cells. Proteins listed on the left were identified based on reactivity ratios exceeding twofold relative to the defined threshold. Heatmaps are ranked by recognition intensity, with the most strongly targeted proteins positioned at the top.

Supplementary figure S3

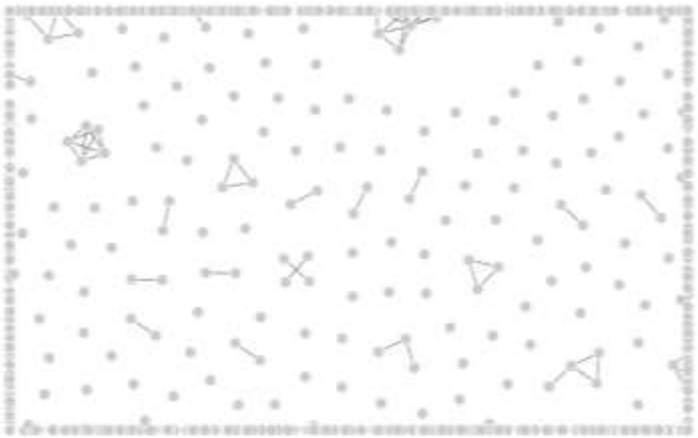

Supplementary Figure S3. Evaluation of pathogen epitope similarity. A total of 948 epitopes were analyzed, corresponding to 942 unique sequences. Clustering was performed using an identity cutoff of 80%, without restrictions on peptide length. All connected clusters were included in the analysis, resulting in the identification of 686 distinct clusters.

Supplementary figure S4

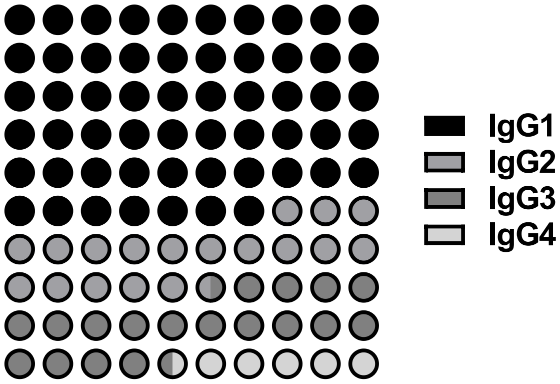

Supplementary Figure S4. Distribution of IgG subclasses in the polyvalent IgG (pIgG) preparation analyzed in this study. The dot plot illustrates the relative abundance of each subclass, expressed as a percentage of the total IgG content in the evaluated formulation.

Supplementary figure S5

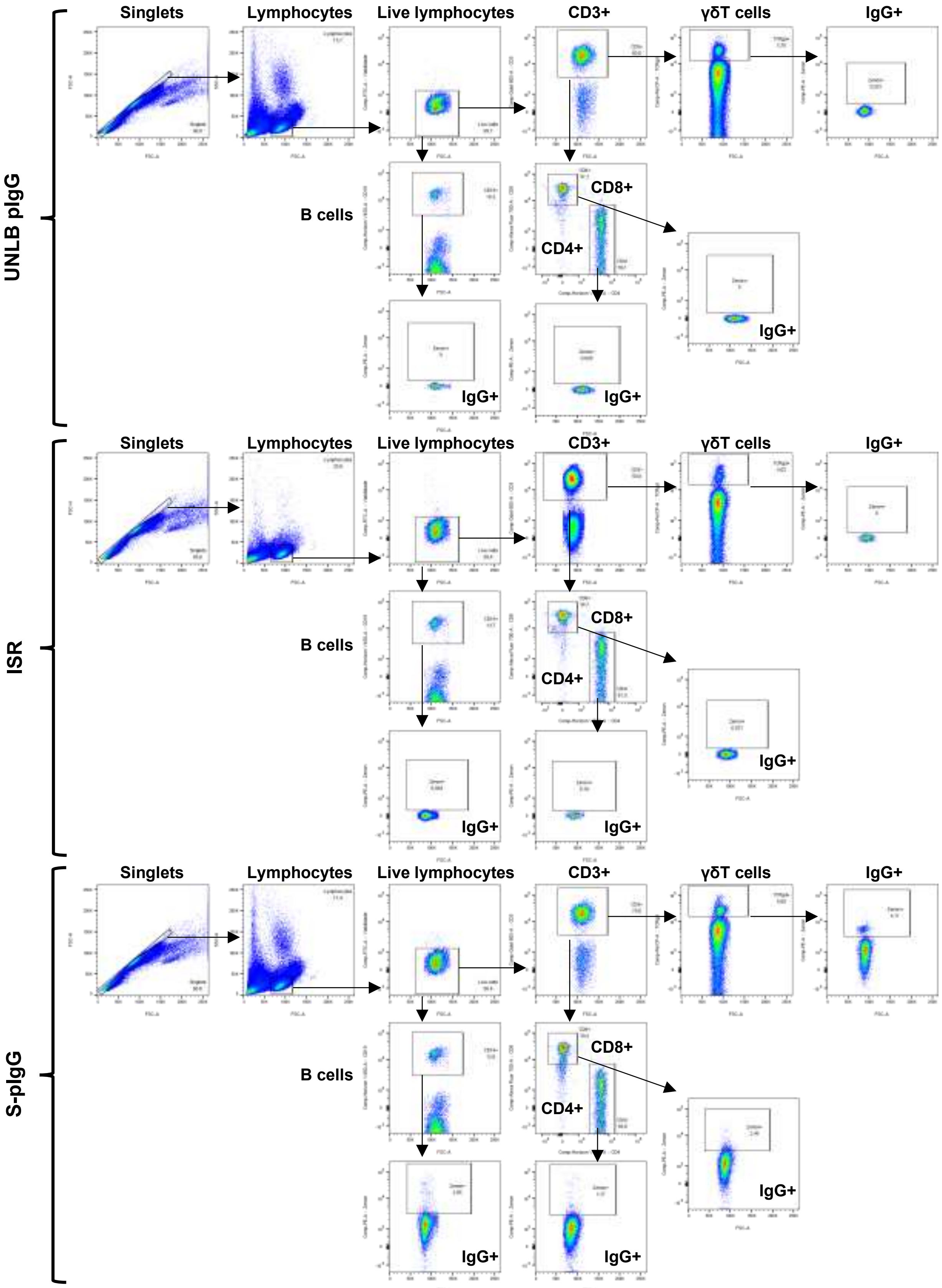

Supplementary Figure S5. Gating strategy used to identify IgG+ (Zenon-stained) CD4+, CD8+, TCRγδ+, and B cells. Arrows indicate the sequential gating steps applied to define each cell population. IgG+ cells are identified in the upper panels, while the lower panels illustrate the corresponding controls: unlabeled pIgG (UNLB pIgG), IgG staining reagents only (ISR), and Zenon-stained pIgG (S-pIgG).

Supplementary figure S6

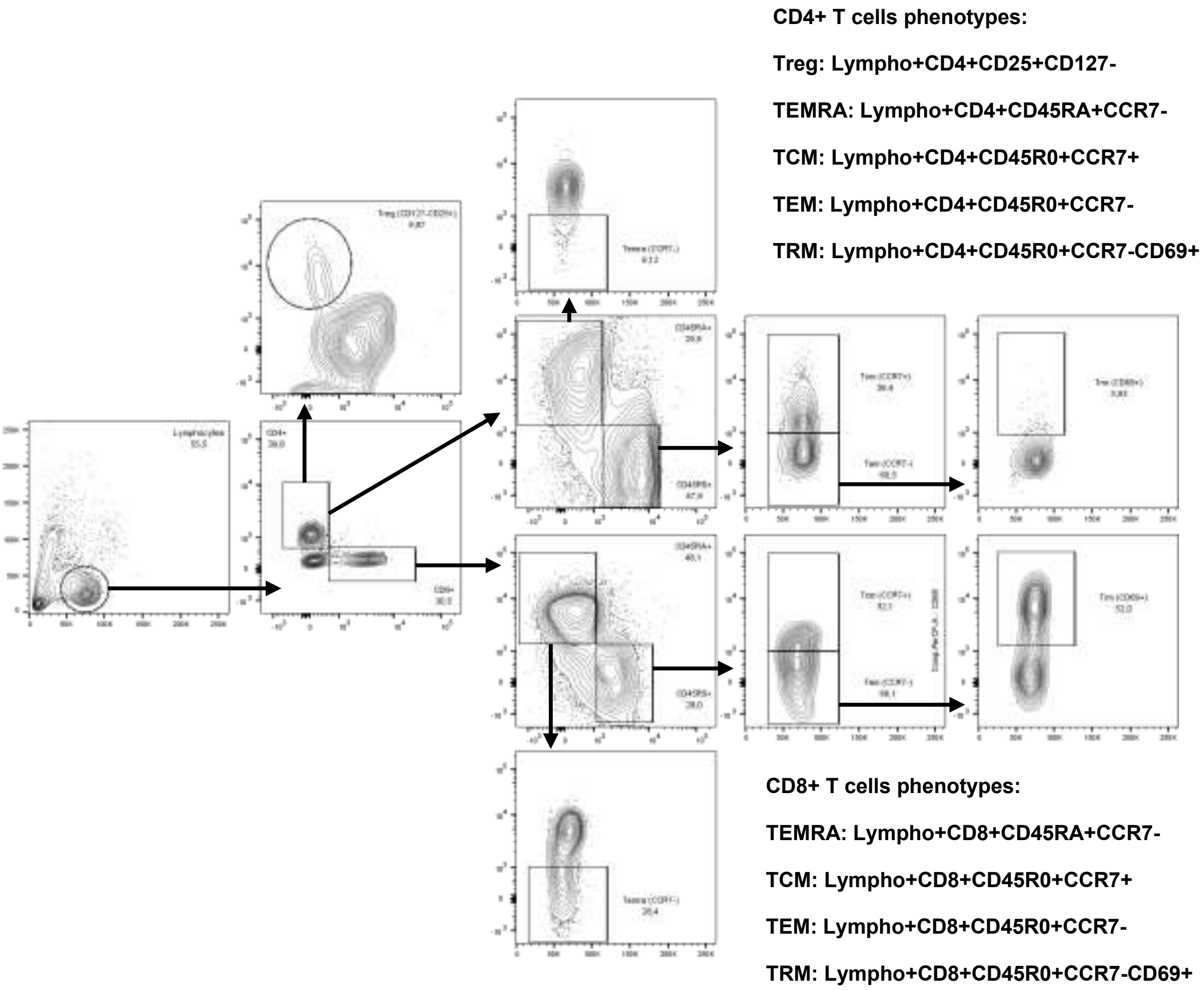

Supplementary Figure S6. Gating strategy used to identify CD4$^+$ and CD8$^+$ T-cell populations. Arrows indicate the sequential gating steps applied to define each phenotype within both CD4$^+$ and CD8$^+$ subsets. Abbreviations: Treg, CD4$^+$ regulatory T cells; TemRA, effector memory T cells re-expressing CD45RA; Tcm, central memory T cells; Tem, effector memory T cells; Trm, tissue-resident memory T cells.
